# Adsorption and desorption characteristics of arsenic onto ceria nanoparticles

**DOI:** 10.1186/1556-276X-7-84

**Published:** 2012-01-23

**Authors:** Qinzhong Feng, Zhiyong Zhang, Yuhui Ma, Xiao He, Yuliang Zhao, Zhifang Chai

**Affiliations:** 1 Key Laboratory of Nuclear Analytical Techniques and Key Laboratory for Biomedical Effects of Nanomaterials and Nanosafety, Institute of High Energy Physics, Chinese Academy of Sciences, Beijing, 100049, China

**Keywords:** ceria nanoparticles, arsenic, adsorption, desorption.

## Abstract

The rapid increase in the use of engineered nanoparticles [ENPs] has resulted in an increasing concern over the potential impacts of ENPs on the environmental and human health. ENPs tend to adsorb a large variety of toxic chemicals when they are emitted into the environment, which may enhance the toxicity of ENPs and/or adsorbed chemicals. The study was aimed to investigate the adsorption and desorption behaviors of arsenic on ceria NPs in aqueous solution using batch technique. Results show that the adsorption behavior of arsenic on ceria NPs was strongly dependent on pH and independent of ionic strength, indicating that the electrostatic effect on the adsorption of these elements was relatively not important compared to surface chemical reactions. The adsorption isotherms fitted very well to both the Langmuir and Freundlich models. The thermodynamic parameters (Δ*H^0^*, Δ*S^0^*, and Δ*G^0^*) for the adsorption of arsenic were determined at three different temperatures of 283, 303, and 323 K. The adsorption reaction was endothermic, and the process of adsorption was favored at high temperature. The desorption data showed that desorption hysteresis occurred at the initial concentration studied. High adsorption capacity of arsenic on ceria NPs suggests that the synergistic effects of ceria NPs and arsenic on the environmental systems may exist when they are released into the environment.

## Introduction

With the large-scale production and widespread application, the potential environmental risks of engineered nanoparticles [NPs] have consequently attracted much attention since NPs will be unavoidably released to the environment after their production, use, and disposal. To date, the potential environmental effects of NPs, in any quantity, are largely unknown [1-3]; therefore, an understanding of the transport, transfer, and fate of NPs in the environment is an important issue for evaluating their environmental and health impacts.

NPs usually exhibit remarkable physical properties, rapid chemical reactivity, and high sorption capacity for inorganic and organic compounds. Studies of the fate and transport of NPs are largely concerned with how their properties and behavior change over time, whether they will interact with toxic contaminants after being released into the environment or they will change the anticipated impact of NPs themselves and the toxic contaminants when they interact with one another. For example, recent studies showed that phenanthrene could be adsorbed by iron and copper NPs through hydrophobic effect and dipole interactions between NPs' charged surface and phenanthrene [4]. Carbon nanotubes [CNTs] tend to adsorb toxic chemicals of atrazine, and CNTs with adsorbed atrazine exhibited the toxicities of both the toxic chemicals and CNTs themselves when interacting with living organisms [5]. Therefore, the potential environmental risks of NPs are exerted not only from the NPs themselves, but also from the toxic contaminants adsorbed by them [4,5]. Hence, knowledge of toxic compound adsorption onto NPs is critical and helpful for their risk assessment, and it is also useful for understanding the effect of nanomaterials on the fate of toxic compounds in the environment [4,5]. Unfortunately, to the best of our knowledge, these studies are limited within zerovalent metal NPs, CNTs, and related materials [4,5]; there are no studies that have examined the adsorption and desorption behaviors of toxic contaminants onto metal oxide NPs.

Ceria NPs are considered to be a representative member of an industrially important class of metal oxide NPs [6,7]; they can be used as automotive catalytic converters [8], UV-blocking agents [9], and single, nanowire-based gas sensors [10]. Indeed, ceria NPs are on the OECD list of priority nanomaterials for immediate testing. However, at present, little ecotoxicity data are available. Previous studies have demonstrated that ceria NPs have toxic effects on cells [11,12], aquatic organisms [13,14], and plants [15]; however, other reports show that ceria NPs are able to rescue HT22 cells from oxidative stress-induced cell death [16] and protect against the progression of cardiac dysfunction [17]. These contradictory results suggest the complex behaviors of ceria NPs in the environment.

Arsenic is one of the most toxic elements and is found virtually in all environmental systems as a result of both geological processes and anthropogenic activities [18,19]; the USEPA has set a maximum contaminant level for arsenic at 10 μg L^-1 ^in drinking water [20]. Adsorption with metal oxides has been widely used to remove these contaminants because of its effectiveness and simplicity for point-of-use applications. However, since the environmental release of ceria NPs from various applications, the NPs' solid surface interactions with organic contaminants, such as arsenic, have become increasingly important, and the subsequent behavior and effects of the released NPs must be considered an urgent need.

Information on the interaction of free and aggregated NPs with adsorbed chemicals is lacking from the literature, although studies suggest that the adsorption of phenanthrene on nanosized zerovalent iron and copper significantly decreases with the increase in pH, that naphthalene exerted significant competition adsorption with phenanthrene [4], and that Th(IV) adsorption onto oxidized MWCNTs is endothermic [21]. The present work investigated the adsorption and desorption behaviors of arsenic onto ceria NPs. The objectives include (1) to quantify and predict the adsorption behavior of arsenic on ceria NPs under different pH and ionic strength, (2) to determine adsorption isotherms and analyze regressively experimental data with Langmuir and Freundlich models, (3) to study the adsorption of arsenic at different temperatures and measure the adsorption thermodynamic parameters (i.e., *ΔH^0^, ΔS^0^, ΔG^0^*), and (4) to investigate the desorption behavior of arsenic at the ceria NPs' surface using batch adsorption experiments.

## Experimental details

### Materials and methods

All chemicals used were of analytical reagent grade. Milli-Q water (18.2 MΩ cm, Millipore, Beijing, China) was used for all solution preparation. Stock solutions of arsenic were purchased from the National Institute of Metrology (Beijing, China). These stock solutions were kept at 4°C in darkness. Working solutions of arsenic (0.4 to 40.0 μg L^-1^) were prepared daily by dilution of the stock solution with HCl. Working solutions of 20% (*w*/*v*) potassium tetrahydroborate were prepared daily by dilution of the KBH_4 _stock solution in 5% NaOH. Pre-reducing solutions containing 10% (*w*/*v*) thiourea (Beijing Chemical Works, Beijing, China) and 10% (*w*/*v*) ascorbic acid (Sigma, Sigma-Aldrich Corporation, St. Louis, MO, USA) were prepared fresh daily in water. Glassware used for the determination of arsenic was soaked in a 10% (*v*/*v*) nitric acid solution and rinsed with Milli-Q water.

### Preparation and characterization of ceria NPs

Ceria NPs used in the experiments were synthesized using a precipitation method as described in our former reports [16,22,23].The resulting ceria NPs were characterized with a Tecnai G^2 ^20 S-Twin transmission electron microscope (FEI Company, Tokyo, Japan) operated at 200 keV and with dynamic light scattering, using a Coulter Nicomp™ 380 ZLS Particle Size Analyzer (Santa Barbara, CA, USA). The surface area was characterized by nitrogen adsorption/desorption analysis of Brunauer-Emmett-Teller (Autosorb-1, Quantachrome Instruments, Boynton Beach, FL, USA).

The X-ray diffraction [XRD] patterns for ceria NPs were recorded on an X' Pert PRO (PANalytical B.V., Almelo, The Netherlands) with monochromatized Cu K_α _radiation. The particle size of CeO_2 _was calculated from the Scherrer formula using the (220) diffraction peak of the respective cerium oxide [24].

### Adsorption experiments

Adsorption isotherms by batch studies were conducted in 7-mL capped centrifuge tubes. Each tube containing 5-mL varying concentrations of arsenic was mechanically shaken on a horizontal motion shaker for 24 h (preliminary experiments had shown that all solid-solution mixtures reached apparent equilibrium within 2 h) at room temperature (25°C). Thermodynamic parameters associated with the adsorption processes were carried out in temperatures of 283, 303, and 323 K. After centrifugation (10,000 rpm for 10 min), the supernatant was sampled and analyzed. The concentration of ceria NPs used for the adsorption experiments was 5 g L^-1^. Initial concentrations of arsenic were ranged from 0.4 to 40.0 μg L^-1^. All experiments were conducted in triplicate.

The solution pH plays a critical role in the adsorption of metalloid ions onto various adsorbents. To optimize the pH for maximum removal efficiency, a sorption experiment was conducted in the initial pH range from 1.0 to 13.0, adjusted using HCl or NaOH.

For ionic strength effect tests, the desired initial sodium nitrate was from 1 × 10^-8 ^to 1.0 mol L^-1^, arsenic was added, and the batch equilibration was initiated immediately. The effect of H_2_SiO_3 _colloid and Fe(OH)_3 _colloid on As(III) and As(V) adsorptions was examined with the concentration from 2 × 10^-6 ^to 0.1 mmol L^-1 ^and from 1 × 10^-7 ^to 0.05 mmol L^-1 ^for H_2_SiO_3 _colloid and Fe(OH)_3 _colloid, respectively.

### Desorption experiments

Desorption experiments of arsenic were carried out in sequential decant-refill steps immediately following the completion of the sorption experiments. The supernatant (4.0 mL) was removed and immediately replaced by the same volume of background solution (0.01 M NaNO_3_), and the vials were resealed and shaken. After 24 h of equilibration, the vials were centrifuged, arsenic in the supernatant was determined, and the amount of arsenic desorbed was calculated from mass differences. The above process was repeated for four complete cycles. All samples were performed at least in triplicate.

### Speciation and analysis of arsenic by hydride generation-atomic fluorescence spectrometry

The AFS-9800 atomic fluorescence spectrometer (Beijing Kechuang Haiguang Instrument Co., Ltd, Beijing, China) was used for arsenic determination. The arsine was generated by adding precise, known volumes of the sample, a reducing solution of potassium tetrahydroborate solution (20%) and hydrochloric acid (10%) using an autosampler. Also, a pre-reducing solution (thiourea 10% and ascorbic acid 10%) was used to pre-reduce arsenic(V) to arsenic(III) when the task was to speciate arsenic(III) and arsenic(V). Arsenic(V) was determined by the difference between total inorganic arsenic and arsenic(III).

## Results and discussion

### Characterization of ceria NPs

**Figure 1:**
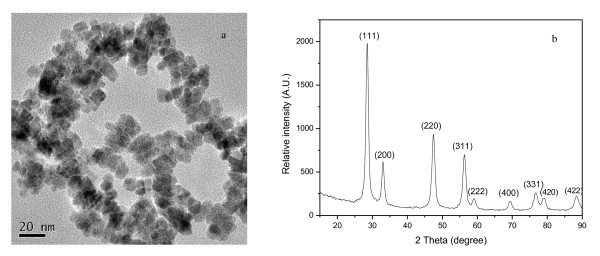
**Characterization of ceria NPs**. (**a**) TEM image of ceria NPs prepared in the present study. (**b**) XRD patterns of ceria NPs powders.

Precipitation method is an attractive, simple, and seemingly general method for producing high-quality ceria NPs under optimum synthesizing conditions. The average particle diameter was 6.6 ± 0.9 nm (Figure 1a). BET analysis demonstrated that the ceria NPs had high specific surface areas which were about 86.85 m^2 ^g^-1^. The size distribution of the hydrodynamic diameter of ceria NPs was about 44.8 ± 1.6 nm.

The results of XRD in Figure 1b showed that ceria NPs were a single-phase, cubic, calcium fluoride-type structure with space group Fm3m, and no evidence was found for the existence of impurities in the products which was consistent with other reports [25].

### Adsorption isotherms

Langmuir and Freundlich isotherms were employed to describe the adsorption of arsenic on ceria NPs at 283, 303, and 323 K. The equation of the Langmuir isotherm is given as Equation 1:

(1) $$\frac{C_{\text{e}}}{q_{\text{e}}}=\frac{1}{q_{\text{max}}K_{\text{L}}}+\frac{C_{\text{e}}}{q_{\text{max}}}.$$

The adsorption capacity *q*_max _and adsorption constant *K*_L _can be determined from the slope and intercept of a linearized plot of *C*_e_/*q*_e _against *C*_e_.

The Freundlich isotherm is derived by assuming a heterogeneous surface with a nonuniform distribution of the heat of sorption over the surface. It can be expressed as follows:

(2) $$q_{\text{e}}=K_{\text{f}}C_{\text{e}}^{1/n}.$$

The equation can be written as a linearized form:

(3) $$\text{ln}\left(q_{\text{e}}\right)=\text{ln}\left(K_{\text{f}}\right)+\left(1/n\right)1\text{n(}{\text{C}}_{\text{e}}),$$

where *K*_f _and *n *are the Freundlich constants: *n *gives an indication of how favorable the adsorption is, and *K*_f _(mg/g (L/mg)^1/*n*^) is the adsorption capacity of the adsorbent. If the value of 1/*n *is lower than 1, it indicates a normal Freundlich isotherm; otherwise, it is indicative of cooperative adsorption. The Freundlich constants can be obtained from the plot of log *q*_e _versus log *C*_e_.

Both Freundlich model and Langmuir model were tested for fitting the arsenic adsorption data. Based on those model equations, the adsorption parameters *q*_max_, *K*_L_, *K*_f_, and *n*, and the correlation coefficient values (*R*) of arsenic onto ceria NPs were obtained (Table 1). As seen in Table 1, the adsorption of arsenic onto ceria NPs was well fitted to the Langmuir isotherm model with high *R *(0.9695 to 0.9815), and this result may be due to the homogeneous distribution of active sites on the surface of ceria NPs. Furthermore, the values of *R*_L _for the Langmuir isotherm were between 0 and 1, and the Freundlich constant 1/*n *was smaller than 1 (0.43 to 0.66), indicating a favorable process. The high adsorption capacity (*q*_max _17.08 to 18.05 mg g^-1^) in the present work reveals that the combination of ceria NPs and arsenic may be efficacious on the environment. The adsorption of arsenic (*q*_max _and *K*_f _in the Langmuir model and the Freundlich model, respectively) increased with the rise in temperature, illuminating an endothermic process for arsenic adsorption onto ceria NPs. The adsorption isotherm was higher at 323 K than the other two isotherms, whereas the adsorption isotherm at 283 K was the lowest. The results indicated that the adsorption of arsenic onto ceria NPs was favored at high temperature and was blocked at low temperature.

**Table 1:** Isotherm model constants of three isotherm models for arsenic adsorption onto ceria NPs

Model	Parameters	Temperature (K)		
		283	303	323
Langmuir model	*q*_max _(mg g^-1^)	17.08	18.02	18.15
	*K*_L _(L mg^-1^)	0.063	0.066	0.24
	*R*	0.9695	0.9704	0.9815
Freundlich model	*K*_f _(mg g^-1^) (L mg^-1^)^1/*n*^	1.32	1.40	2.32
	*n*	1.54	1.52	1.77
	*R*	0.9957	0.9948	0.9966

*q*_max_, adsorption capacity; *K*_L_, adsorption constant; *K*_f _and *n*, Freundlich constants; *R*, correlation coeeficient.

### Thermodynamic parameters associated with the adsorption processes

The thermodynamic adsorption parameters can be calculated from the variation of the thermodynamic equilibrium constant *K_0 _*under different temperatures. The standard molar free energy of adsorption [*ΔG^0^*] is calculated from the relationship:

(4) $$\Delta G^{0}=-\text{RT ln}K_{0}.$$

The average standard enthalpy [*ΔH^0^*] is obtained from the slope of the linear variation of ln*K_0 _*versus 1/T.

(5) $$\text{ln}K_{0}=-\frac{\Delta H^{0}}{\text{RT}}+\text{constant}\text{.}$$

The standard entropy [*ΔS^0^*] is calculated from:

(6) $$\Delta S^{0}=\left(\Delta H^{0}-\Delta G^{0}\right)/T.$$

The thermodynamic adsorption parameter values obtained were given in Table 2. The adsorption enthalpy of arsenic onto ceria NPs was 17.89 kJ mol^-1^, indicating that the adsorption process of arsenic onto ceria NPs was endothermic. It was reported that the enthalpy of physisorption was smaller than 20 kJ mol^-1 ^[22]. Based on the above Δ*H^0^*, it suggests that the adsorption of arsenic onto ceria NPs is a physisorption process. The positive adsorption entropy indicated that the degrees of freedom increased at the solid-liquid interface during the adsorption of arsenic onto ceria NPs. *ΔG^0 ^*values were in the range of -1.40 to -4.18 kJ mol^-1 ^when the adsorption temperature was from 283 to 323 K, and the negative values of *ΔG^0 ^*indicated that the adsorption process was spontaneous. The thermodynamic adsorption data indicated that the adsorption of arsenic onto ceria NPs was an endothermic process, and the process was favorable at high temperature. The results were consistent with the adsorption isotherm results.

**Table 2:** Thermodynamic parameters for arsenic adsorption onto ceria NPs

Temperature (K)	Thermodynamic parameters			
	ln(*K*_0_)	Δ*G*^0 ^(kJ mol^-1^)	Δ*H*^0 ^(kJ mol^-1^)	Δ*S*^0 ^(J mol^-1 ^K^-1^)
283	0.59	-1.40	17.89	71.52
303	0.65	-1.65	17.89	70.51
323	1.56	-4.18	17.89	80.78

Δ*G*^0^, standard molar free energy of adsorption; Δ*H*^0^, average standard enthalpy; Δ*S*^0^, standard entropy.

### Adsorption and desorption

**Figure 2:**
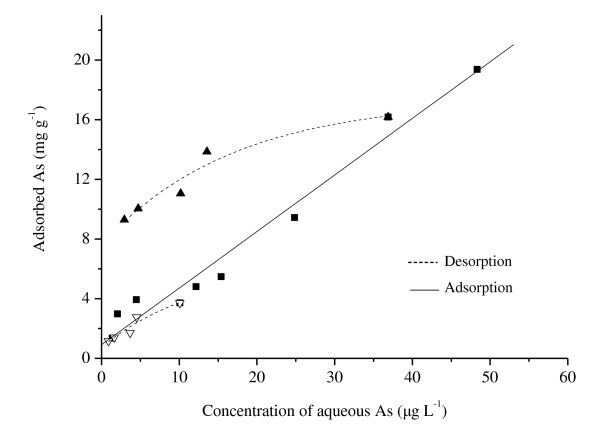
**Adsorption and desorption isotherms of arsenic on ceria NPs**. Filled square, adsorption data; filled triangle, 80 μg L^-1 ^of initial arsenic with desorption; empty inverted triangle, 20 μg L^-1 ^of initial arsenic with desorption; *T *= 298 K.

Figure 2 shows the adsorption and desorption isotherms of arsenic on ceria NPs. There would be a corresponding concentration-dependent effect in this study: the desorption of ceria NPs had no significant desorption hysteresis for 20 μg L^-1 ^of arsenic, while it had significant desorption hysteresis when the initial concentration of arsenic was 80 μg L^-1 ^with the thermodynamic index of irreversibility ranging from 0.58 to 0.95.

Hysteresis is referred to as irreversible adsorption which is reproducible and originates from a shift in the equilibrium point when a reaction approaches equilibrium from desorption direction rather than sorption [4,26,27]. Similar result was reported by Shirvani et al. that the quantity of Cd desorbed from the minerals after five cycles of desorption was depending on the initial load of Cd on the minerals [26]. Recent studies supported irreversible deformation as the cause of hysteresis in flexible solids, such as natural organic matter and organoclays [5,27]. During the adsorption-desorption cycle, pore deformation may result in some of adsorbent transferred to sites in the solid where free exchange with molecules in the bulk fluid phase is no longer possible.

Desorption of inorganic and organic contaminants from the adsorbent is rapidly gaining recognition owing to its importance to the fate, toxicity, and transport of these contaminants in the environmental system. If the contaminants would be released from the adsorbent in significant concentrations, either in the environmental or biotic mediums, they would be a risk for living organisms. In this work, high adsorption capacity and reversible adsorption of arsenic on ceria NPs suggest that ceria NPs may influence the fate and behavior of arsenic in the environment. Thus, ceria NPs with arsenic may exhibit synergistic effects when interacting with living organisms.

### Adsorption characteristics of arsenic(III) and arsenic(V) in different environmental conditions

Inorganic arsenic has four oxidation states: +5, +3, 0, and -3. In the soil/water environment, it is mainly present in the +3 and +5 oxidation states. In reduced environments, arsenious acid is a common arsenic(III) aqueous species, whereas oxidized environments contain more arsenic(V) aqueous species. These two aqueous species may adsorb onto inorganic and organic components and precipitate in a variety of forms. The environmental fate of arsenic in subsurface environments is highly dependent on the arsenic speciation, pH, ionic strength, and the presence of adsorbents such as metal oxides. Furthermore, there are many kinds of colloidal solutions with different charges in water, for example, in general, H_2_SiO_3 _colloid surface has a negative charge and Fe(OH)_3 _colloid surface has a positive charge, which may affect the adsorption of arsenic on ceria NPs. However, little investigation had been conducted on the adsorption of arsenic by ceria NPs in different colloidal solutions. The experiments below were carried out to investigate the adsorption characteristic of arsenic onto ceria NPs in different environmental conditions.

**Figure 3:**
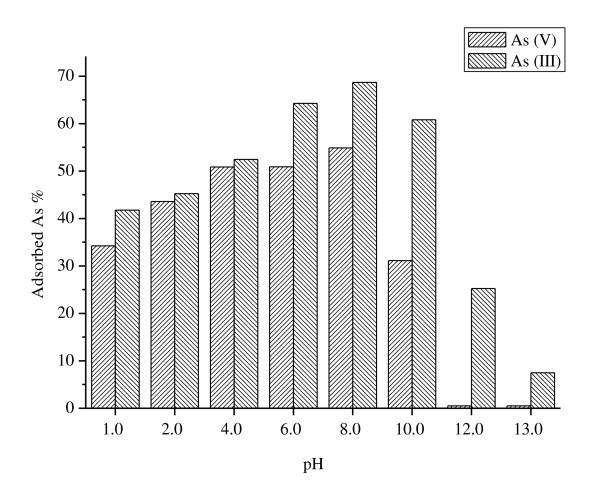
**Effect of pH on the adsorption of As(III) and As(V) on ceria NPs**. *C*_initial arsenic _= 80 μg L^-1^, *I *= 0.01 mol L^-1 ^NaNO_3_, *T *= 298 K.

Both arsenate and arsenite adsorptions on ceria NPs as a function of solution pH exhibited a parabolic adsorption curve which was showed in Figure 3. Adsorption increased with increasing solution pH from pH 1 to 6 and then decreased with increasing pH from pH 6 to 13 for arsenate, which was similar to the report that arsenate adsorption on the clays increased with increasing solution pH from pH 3 to 5 and decreased with increasing solution pH from pH 5 to 9 [28]. As for arsenite, adsorption increased with increasing solution pH from pH 1 to 8 (the maximum adsorption was 70%) and then decreased with increasing pH from pH 8 to 13; a similar behavior was found for arsenite adsorption on the clays and amorphous Al oxide [28]. The maximum adsorption of arsenite occurs in the weak alkaline region, up to pH 10; OH^- ^competition for the arsenite centers at high pH values which leads to the decreasing adsorption.

From Figure 3, it could also be found that arsenite is commonly more strongly adsorbed on ceria NPs than arsenate especially when pH was above 9. Theoretically, arsenate is commonly more strongly adsorbed on ceria NPs than arsenite since arsenate presents as H_2_AsO_4_^- ^and HAsO_4_^2- ^when pH is from 2 to 8, while arsenite presents as As(OH)_3_^0 ^species. Some reports showed that at all pH values, arsenic(V) was more strongly bound than arsenic(III) [28-30]. Thus, further experiments in our future study should be carried out to study the adsorption mechanism.

**Figure 4:**
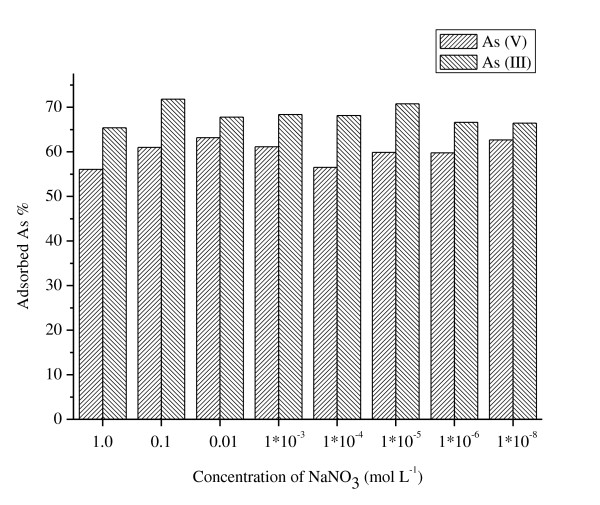
**Effect of ion strength on the adsorption of As(III) and As(V) on ceria NPs**. *C*_initial arsenic _= 80 μg L^-1^, *T *= 298 K.

The effects of ionic strength on the adsorption of arsenic(III) and arsenic(V) onto the surfaces of ceria NPs were presented in Figure 4. The amount of arsenic(III) and arsenic(V) adsorbed by ceria NPs was stable with increasing ionic strength from 1 × 10^-8 ^to 1.0 mol L^-1 ^NaNO_3_. Therefore, ionic strength generally had very minor or no impact on the adsorption in this study. Similar results were also observed by other reports when iron oxide or other sorbents were used [31-35].

The ionic strength can influence the double-layer thickness and interface potential; thereby, it can affect the binding of the sorbed species [36,37]. The specific and nonspecific adsorptions can be distinguished by evaluating the effect of ionic strength on anion partitioning [37]. It was reported that the specific adsorption is unaffected by the change in ionic strength, whereas the nonspecific adsorption is likely to be influenced greatly by the change of ionic strength because of the competitive adsorption with counteractions. The insignificant impact of ionic strength indicated that the specific adsorption was found on the adsorption of arsenic(V) and arsenic(III) by ceria NPs which resulted from an inner sphere complex formation between arsenic and the surface of ceria NPs. The strong pH dependence and ionic strength independence further indicate that the adsorption mechanism is mainly surface complexation rather than ion exchange.

**Figure 5:**
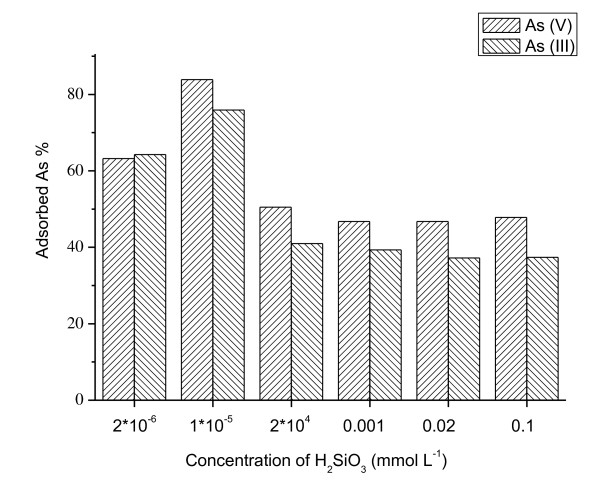
**Effect of H_2_SiO_3 _colloid on the adsorption of As(III) and As(V) on ceria NPs**. *C*_initial arsenic _= 80 μg L^-1^, *T *= 298 K.

H_2_SiO_3 _colloid was homemade with the particle size of 50 nm. Figure 5 reveals that arsenic adsorption on ceria NPs increased significantly with the increasing of H_2_SiO_3 _colloid concentration from 2 × 10^-6 ^to 1 × 10^-5 ^mmol L^-1^, while it was stable when H_2_SiO_3 _colloid concentration was from 2 × 10^-4 ^to 0.1 mmol L^-1^. Meanwhile, ceria NPs had lower adsorption capacity for As(III) than for As(V) with the existence of H_2_SiO_3 _colloidal solution. The possible reason may be the different pH in these solutions. When the concentrations of H_2_SiO_3 _colloidal solution were lower than 0.001 mol L^-1^, the pH values were from 5.35 to 5.91. Acid dissociation [p*K*_a_] values for arsenious acid are as follows: p*K*_a_^1 ^= 9.2 and p*K*_a_^2 ^= 12.1, while p*K*a values for arsenic acid are p*K*_a_^1 ^= 2.3, p*K*_a_^2 ^= 6.8, and p*K*_a_^3 ^= 11.6. Therefore, at pH 5.35 to 5.91, the surface has a net positive charge; As(III) exists in neutral H_3_AsO_3_^0 ^which has less influence than the adsorption for negatively charged As(V) species which exists in H_2_AsO_4_^-^. The inflections or maxima in the adsorption of arsenic at pH values close to their p*K*_a _values are a well-documented phenomenon [38,39].

**Figure 6:**
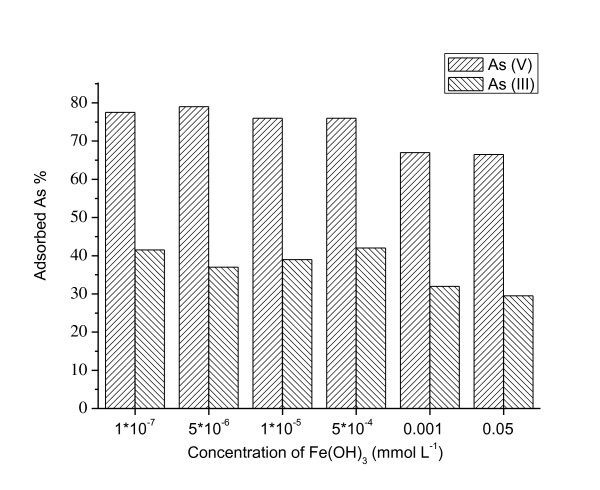
**Effect of Fe(OH)_3 _colloid on the adsorption of As(III) and As(V) on ceria NPs**. *C*_initial arsenic _= 80 μg L^-1^, *T *= 298 K.

Fe(OH)_3 _colloidal solution was synthesized in our laboratory, and TEM showed that Fe(OH)_3 _particle was a nanorod with 30 to 70 nm in long diameter. NPs with a high specific surface area tend to adsorb a large variety of toxic chemicals, which may enhance the toxicity of toxic chemicals. It was found from Figure 6 that arsenic adsorption on ceria NPs increased stably with the increasing of H_2_SiO_3 _colloid concentration from 1 × 10^-7 ^to 0.05 mmol L^-1^; furthermore, ceria NPs had lower As(III) adsorption than As(V) adsorption under these conditions. Such a difference in As(III) and As(V) sorption efficiencies on ceria NPs could be related to possible differences between arsenic surface complexes on these different absorbents. Interestingly, a recent study by Wang et al. [40] proposed that arsenate is absorbed more efficiently than arsenite on green rusts: for arsenate, the presence of binuclear bidentate double-corner complexes and mononuclear monodentate corner-sharing complexes at the surface of green rusts, and for arsenite, the presence of dimers of As(III) pyramids binding to the edges of the GR1Cl layers by corner sharing with FeO_6 _octahedra. On the other hand, Raven et al. [39] observed that arsenite adsorption was higher than arsenate adsorption on ferrihydrite throughout the pH range of 3 to 11. These findings indicate that different ferrous-based compounds have different adsorption capacities for arsenic, which in turn complicates the adsorption behavior of arsenic in ceria NPs.

## Conclusions

The high adsorption capacity and reversible adsorption of arsenic on ceria NPs clearly confirm that ceria NPs can interact with arsenic when they are released into the environment, which may influence the fate and behavior of arsenic and may enhance the toxicity of ENPs and/or arsenic. Taken together, the results indicate that studies dealing with the potential ecotoxicological effects of NPs for the environment have to be designed carefully in order to have the defined understanding between the NPs and the adsorbed chemicals in the environment.

## Competing interests

The authors declare that they have no competing interests.

## Authors' contributions

QF designed the study, performed the research, and prepared the manuscript. ZZ provided the experimental idea and advised the experimental design. YM synthesized the target material and performed the statistical analysis. XH participated in the adsorption experiments. YZ and ZC contributed to the experimental idea and drafted the manuscript. All authors read and approved the final manuscript.

## References

[B1] Stephen JK Pedro JJ Graeme EB Teresa FF Richard DH Delina YL Shaily M Michael JM Jamie RL Nanomaterials in the environment: behavior, fate, bioavailability, and effects Environ Toxicol Chem 2008 27 1825 2851 10.1897/08-090.1 19086204

[B2] Grazyna B Jerzy G Pawel LU Nanoparticles: their potential toxicity, waste and environmental management Waste Manage 2009 29 2587 2595 10.1016/j.wasman.2009.04.001 19427190

[B3] Moore MN Do nanoparticles present ecotoxicological risks for the health of the aquatic environment? Environ Int 2006 32 967 976 10.1016/j.envint.2006.06.014 16859745

[B4] Fang J Shan XQ Wen B Lin JM Lu XC Liu XD Owens G Sorption and desorption of phenanthrene onto iron, copper, and silicon dioxide nanoparticles Langmuir 2008 24 10929 10935 10.1021/la801459s 18729339

[B5] Yan XM Shi BY Lu JJ Feng CH Wang DS Tang HX Adsorption and desorption of atrazine on carbon nanotubes J Colloid Interf Sci 2008 321 30 38 10.1016/j.jcis.2008.01.047 18294649

[B6] Birbaum K Brogioli R Schellenberg M Martinoia E Stark WJ Günther D Limbach LK No evidence for cerium dioxide nanoparticle translocation in maize plants Environ Sci Technol 2010 44 8718 8723 10.1021/es101685f 20964359

[B7] Ludwig KL Robert B Elisabeth M Rolf K René G Wendelin JS Removal of oxide nanoparticles in a model wastewater treatment plant: influence of agglomeration and surfactants on clearing efficiency Environ Sci Technol 2008 42 5828 5833 10.1021/es800091f 18754516

[B8] Chen JP Patil S Seal S Mcginnis JF Rare earth nanoparticles prevent retinal degeneration induced by intracellular peroxides, nature nanotechnology Nature Nanotechnology 2006 1 142 150 10.1038/nnano.2006.91 18654167

[B9] Zholobak NM Ivanov VK Shcherbakov AB Shaporev AS Polezhaeva OS Baranchikov AY Spivak NY Tretyakov YD UV-shielding property, photocatalytic activity and photocytotoxicity of ceria colloid solutions J Photoch Photobio B 2010 102 32 38 10.1016/j.jphotobiol.2010.09.002 20926307

[B10] Yuan Q Duan HH Li LL Sun LD Zhang YW Yan CH Controlled synthesis and assembly of ceria-based nanomaterials J Colloid Interf Sci 2009 335 151 167 10.1016/j.jcis.2009.04.007 19439316

[B11] Thill A Zeyons O Spalla O Chauvat F Rose J Auffan M Flank AM Cytotoxicity of CeO2 nanoparticles for *Escherichia coli*. Physico-chemical insight of the cytotoxicity mechanism Environ Sci Technol 2006 40 6151 6156 10.1021/es060999b 17051814

[B12] Safi M Sarrouj H Sandre O Mignet N Berret JF Interactions between sub-10-nm iron and cerium oxide nanoparticles and 3T3 fibroblasts: the role of the coating and aggregation state Nanotechnology 2010 21 145103 10.1088/0957-4484/21/14/145103 20234082

[B13] Hoecke KV Quik JT Mankiewicz-Boczek J Schamphelaere KA Elsaesser A Meeren PV Barnes C McKerr G Howard VC Meent DV Rydzyński K Dawson KA Salvati A Lesniak A Lynch I Silversmit G Samber BD Vincze L Janssen CR Fate and effects of CeO_2 _nanoparticles in aquatic ecotoxicity tests Environ Sci Technol 2009 43 4537 4546 10.1021/es9002444 19603674

[B14] Johnston BD Scown TM Moger J Cumberland SA Baalousha M Linge K Aerle R Jarvis K Lead JR Tyler CR Bioavailability of nanoscale metal oxides TiO_2_, CeO_2_, and ZnO to fish Environ Sci Technol 2010 44 1144 1151 10.1021/es901971a 20050652

[B15] López-Moreno ML Rosa G Hernández-Viezcas J Castillo-Michel H Botez CE Peralta-Videa JR Gardea-Torresdey JL Evidence of the differential biotransformation and genotoxicity of ZnO and CeO_2 _nanoparticles on soybean (*Glycine max*) plants Environ Sci Technol 2010 44 7315 7320 10.1021/es903891g 20384348 PMC2944920

[B16] Lu K Zhang ZY He X Ma YH Zhou KB Zhang HF Bai W Ding YY Wu ZQ Zhao YL Chai ZF Bioavailability and distribution of ceria nanoparticles in simulated aquatic ecosystems, quantification with a radiotracer technique J Nanosci Nanotechno 2010 10 8658 8662 10.1166/jnn.2010.2494 21121379

[B17] Niu JL Asim A Linda MR Wang XH Pappachan EK Cardioprotective effects of cerium oxide nanoparticles in a transgenic murine model of cardiomyopathy Cardiovasc Res 2007 73 549 559 10.1016/j.cardiores.2006.11.031 17207782 PMC1855085

[B18] Huang JH Ilgen G Vogel D Michalzik B Hantsch S Tennhardt L Bilitewski B Emissions of inorganic and organic arsenic compounds via the leachate pathway from pretreated municipal waste materials: a landfill reactor study Environ Sci Technol 2009 43 7092 7097 10.1021/es901605q 19806747

[B19] Williams PN Lei M Sun GX Huang Q Lu Y Deacon C Meharg AA Zhu YG Occurrence and partitioning of cadmium, arsenic and lead in mine impacted paddy rice: Hunan, China Environ Sci Technol 2009 43 637 642 10.1021/es802412r 19244995

[B20] Arai Y Lanzirotti A Sutton S Davis JA Sparks DL Arsenic speciation and reactivity in poultry litter Environ Sci Technol 2003 37 4083 4090 10.1021/es0340580 14524439

[B21] Chen CL Li XL Zhao DL Tan XL Wang XK Adsorption kinetic, thermodynamic and desorption studies of Th(IV) on oxidized multi-wall carbon nanotubes Colloids Surface A 2007 302 449 454 10.1016/j.colsurfa.2007.03.007

[B22] Zhang F Jin Q Chan SW Ceria nanoparticles: size, size distribution, and shape J Appl Phys 2004 95 4319 4325 10.1063/1.1667251

[B23] Ma YH Kuang LL He X Bai W Ding YY Zhang ZY Zhao YL Chai ZF Effects of rare earth oxide nanoparticles on root elongation of plants Chemosphere 2010 78 273 279 10.1016/j.chemosphere.2009.10.050 19897228

[B24] Zhang J Ju X Wu ZY Liu T Hu TD Xie YN Structural characteristics of cerium oxide nanocrystals prepared by the microemulsion method Chem Mater 2001 13 4192 4197 10.1021/cm010235p

[B25] Rane N Zou H Buelna G Lin JYS Sol-gel synthesis and properties of unsupported and supported mesoporous ceria membranes J Membrane Sci 2005 256 89 97

[B26] Shirvani M Kalbasi M Shariatmadari H Nourbakhsh F Najafi B Sorption-desorption of cadmium in aqueous palygorskite, sepiolite, and calcite suspensions: isotherm hysteresis Chemosphere 2006 65 2178 2184 10.1016/j.chemosphere.2006.06.002 16870231

[B27] Sander M Lu YF Pignatello JJ A thermodynamically based method to quantify true sorption hysteresis J Environ Qual 2005 34 1063 1072 10.2134/jeq2004.0301 15888892

[B28] Goldberg S Competitive adsorption of arsenate and arsenite on oxides and clay minerals sabine goldberg Soil Sci Soc Am J 2002 66 413 421 10.2136/sssaj2002.0413

[B29] Chakraborty S Wolthers M Chatterjee D Charlet L Adsorption of arsenite and arsenate onto muscovite and biotite mica J Colloid Interf Sci 2007 309 392 401 10.1016/j.jcis.2006.10.014 17292378

[B30] Buschmann J Kappeler A Lindauer U Kistler D Berg M Sigg L Arsenite and arsenate binding to dissolved humic acids: influence of pH, type of humic acid, and aluminum Environ Sci Technol 2006 40 6015 6020 10.1021/es061057+ 17051793

[B31] Antelo J Avena M Fiol S López R Arce F Effects of pH and ionic strength on the adsorption of phosphate and arsenate at the goethite-water interface J Colloid Interf Sci 2005 285 476 486 10.1016/j.jcis.2004.12.032 15837462

[B32] Vithanage M Chandrajith R Bandara A Weerasooriya R Mechanistic modeling of arsenic retention on natural red earth in simulated environmental systems J Colloid Interf Sci 2006 294 265 272 10.1016/j.jcis.2005.07.026 16146634

[B33] Arai Y Elzinga EJ Sparks DL X-ray absorption spectroscopic investigation of arsenite and arsenate adsorption at the aluminum oxide-water interface J Colloid Interf Sci 2001 235 80 88 10.1006/jcis.2000.7249 11237445

[B34] Catalano JG Zhang Z Fenter P Bedzyk MJ Inner-sphere adsorption geometry of Se(IV) at the hematite (100)-water interface J Colloid Interf Sci 2006 297 665 671 10.1016/j.jcis.2005.11.026 16386265

[B35] Su TZ Guan XH Gu GW Wang JM Adsorption characteristics of As(V), Se(IV), and V(V) onto activated alumina: effects of pH, surface loading, and ionic strength J Colloid Interf Sci 2008 326 347 353 10.1016/j.jcis.2008.07.026 18706566

[B36] Chen CL Wang XK Sorption of Th (IV) to silica as a function of pH, humic/fulvic acid, ionic strength, electrolyte type Appl Radiat Isotopes 2007 65 155 163 10.1016/j.apradiso.2006.07.003 17142051

[B37] Xu RK Wang Y Tiwari D Wang HY Effect of ionic strength on adsorption of As(III) and As(V) on variable charge soils J Environ Sci 2009 21 927 932 10.1016/S1001-0742(08)62363-3 19862958

[B38] Altundoğan HS Altundoğan S Tümen F Bildik M Arsenic adsorption from aqueous solutions by activated red mud Waste Manage 2002 22 357 363 10.1016/S0956-053X(01)00041-1 11952183

[B39] Raven K Jain A Loeppert R Arsenite and arsenate adsorption on ferrihydrite: kinetics, equilibrium, and adsorption envelopes Environ Sci Technol 1998 32 344 349 10.1021/es970421p

[B40] Wang YH Morin G Ona-Nguema G Juillot F Guyot F Calas G Brown GE Evidence for different surface speciation of arsenite and arsenate on green rust: an EXAFS and XANES Study Environ Sci Technol 2010 44 109 115 10.1021/es901627e 20039740

